# Antibody Microarray for *E. coli* O157:H7 and Shiga Toxin in Microtiter Plates

**DOI:** 10.3390/s151229807

**Published:** 2015-12-04

**Authors:** Andrew G. Gehring, Jeffrey D. Brewster, Yiping He, Peter L. Irwin, George C. Paoli, Tawana Simons, Shu-I Tu, Joseph Uknalis

**Affiliations:** Molecular Characterization of Foodborne Pathogens Research Unit, United States Department of Agriculture-Northeast Area, Agricultural Research Service, Eastern Regional Research Center, Wyndmoor, PA 19038, USA; jeffrey.brewster@ars.usda.gov (J.B.); yiping.he@ars.usda.gov (Y.H.); peter.irwin@ars.usda.gov (P.I.); george.paoli@ars.usda.gov (G.P.); tawana.simons@ars.usda.gov (T.S.); shui.tu@ars.usda.gov (S.-I.T.); joseph.uknalis@ars.usda.gov (J.U.)

**Keywords:** antibody microarray, bacteria, centrifugation, fluorescence, immunoassay, multiwell, microtiter plate, multiplex, toxin

## Abstract

Antibody microarray is a powerful analytical technique because of its inherent ability to simultaneously discriminate and measure numerous analytes, therefore making the technique conducive to both the multiplexed detection and identification of bacterial analytes (*i.e*., whole cells, as well as associated metabolites and/or toxins). We developed a sandwich fluorescent immunoassay combined with a high-throughput, multiwell plate microarray detection format. Inexpensive polystyrene plates were employed containing passively adsorbed, array-printed capture antibodies. During sample reaction, centrifugation was the only strategy found to significantly improve capture, and hence detection, of bacteria (pathogenic *Escherichia coli* O157:H7) to planar capture surfaces containing printed antibodies. Whereas several other sample incubation techniques (e.g., static *vs*. agitation) had minimal effect. Immobilized bacteria were labeled with a red-orange-fluorescent dye (Alexa Fluor 555) conjugated antibody to allow for quantitative detection of the captured bacteria with a laser scanner. Shiga toxin 1 (Stx1) could be simultaneously detected along with the cells, but none of the agitation techniques employed during incubation improved detection of the relatively small biomolecule. Under optimal conditions, the assay had demonstrated limits of detection of ~5.8 × 10^5^ cells/mL and 110 ng/mL for *E. coli* O157:H7 and Stx1, respectively, in a ~75 min total assay time.

## 1. Introduction

According to the U.S. Centers for Disease Control and Prevention, approximately 48 million illnesses; 128,000 hospitalizations; and 3000 deaths per year in the United States alone are attributed to ingestion of contaminated foods [1]. Traditional microbial culture methods can detect and identify a single, specific bacterium contaminant in foods, but the approach may require days or weeks to complete and typically, quantitative data is not generated. Such specific detection of very small numbers (e.g., 1 cell/mL) of pathogenic bacteria in complex food matrices necessitates methods with extremely high sensitivity. The quest for faster assay times (of minutes to hours) combined with quantitative, low level detection results has stimulated the development of rapid microbial methods, many of which are biosensor based [2,3,4].

A notorious bacterial pathogen, *Escherichia coli* O157:H7, can cause severe sickness (e.g., hemorrhagic colitis and hemolytic uremic syndrome) and death for some infected by the microorganism [5]. Sickness associated with foodborne *E. coli* O157:H7 is an important problem in the United States where past multistate outbreaks have been associated with meat [6] and produce [7]. *E. coli* O157:H7 is classified as a “zero tolerance” adulterant and is therefore perceived as a major concern due to the threat of incidental contamination of foods with the pathogen. Therefore, considerable effort has been undertaken to develop specific, rapid methods for the detection of pathogens associated with foodborne outbreaks [2,8,9].

Rapid methods with the capacity to screen for analytes of differing size (e.g., ranging from biomolecular toxins to whole bacterial cells) can be useful for multivariate analysis [10]. In addition, the desire to screen large numbers of samples for reliable food safety monitoring necessitates high-throughput analytical processing. Nucleic acid microarrays have exhibited enormous potential for pathogen screening [11,12]. Similarly, protein microarrays comprised of antibodies as biorecognition elements orthogonally arrayed in spots or parallel printed stripes have also been generated for the detection and typing of pathogens. Several examples of antibody arrays that show promise for the multiplex detection of bacterial cells and/or toxins in complex sample matrices (e.g., foods) have been developed [13,14,15,16,17], as well as commercialized [18]. The evolution, application, and merits of antibody, or protein, microarrays have been reviewed elsewhere [19,20,21,22,23,24].

Past research in this group has demonstrated the high-throughput and multiplex capability of antibody microarray in multiwell format [15]. This study presents a streamlined and improved version of that system with an optimized assay that considerably reduces the overall assay time with a concomitantly better limit of detection (LOD) for bacterial cells. A “bottleneck” in improvement of LOD has been that bacteria suspended in aqueous medium are relatively immobile in part due to their density being essentially that of water. Hence, under static incubation conditions, non-flagellated and/or dead bacteria (essentially large “particles”) that may exhibit Brownian motion travel an insignificant distance when suspended in aqueous medium. Even the metabolic-dependent motion of flagellated bacteria is quite slow [25]. Therefore, under their own accord, most bacteria suspended in bulk solvent do not come in close contact with planar binding surfaces, which, in this study, was passively adsorbed with capture antibodies to relatively inexpensive polystyrene plate well bottoms that served as microarray substrates. At low concentrations (≤10^6^/mL), the cells are relatively dispersed so that binding events are rare. Increased assay sensitivity necessitates improved antibody-based immobilization of bacteria to solid supports. Dielectrophoresis [26] and direct radiation force combined with ultrasound acoustic streaming [27] have been employed as means to improve immobilization via active partitioning of bacteria from liquid phase to static, antibody-coated, solid substrates. Other groups, such as Ball *et al*. [28], have employed centrifugation to mechanically force bacteria to a capture surface. Our efforts focused on the latter technique given its simplicity, rapidity, and particularly its availability for immediate application with our transparent, polystyrene array substrates. A major part of this investigation compared the efficacy (*i.e*., increased fluorescence responses associated with bacterial capture) of binding for bacteria (*E. coli* O157:H7) versus the biomolecule, Shiga toxin 1 (Stx1; a protein synthesis inhibitor that is produced by Shigatoxigenic strains of *E. coli*), at the capture surface of a microarray substrate as influenced by various incubation conditions (static, mixing, and centrifugation).

## 2. Experimental Section

### 2.1. Materials

Reagents used in this research were: glycerol, tablets of phosphate-buffered saline (PBS; 10 mM phosphate, 2.7 mM KCl, 137 mM NaCl, pH 7.4), fraction V bovine serum albumin (BSA) from Sigma (St. Louis, MO, USA) and NeutrAvidin from Thermo Scientific (Waltham, MA, USA). Plates used were MicroAmp^®^ 384-well reaction/microarray source plates (polypropylene, conical wells) from PE Biosystems (Carlsbad, CA, USA) and antibodies were printed on black-walled, clear/transparent and flat-bottomed, polystyrene 96-multiwell microtiter/microarray destination plates (well dimensions—6.6 mm diameter, ~11 mm height) with (FLUOTRAC 600) surfaces from Greiner Bio-One North America Inc. (Monroe, NC, USA). Anti-*E. coli* O157:H7 antibody (unmodified or biotinylated; polyclonal IgG affinity purified for target, exclusivity purified against non-target *E. coli* strains) raised in goats was obtained from Kirkegaard and Perry Laboratories, Inc. (Gaithersburg, MD, USA). Alexa Fluor 555 (AF555) dye labeling kit (from Invitrogen, Carlsbad, CA USA) was used to prepare fluorescent BSA and antibody conjugates. Stx1 and anti-Stx1 antibody solution comprised of equal parts of 9C9 (IgG_1_; A, A_1_, B neutralizing), 10D11 (IgG_2_b; A, A_1_, B neutralizing), and 13C4 (IgG_1_κ; B neutralizing) murine monoclonal antibodies initially constituted in 50% glycerol in nH_2_O (employed for analyte capture) and 3C10 (IgG_1_; A, A_1_, B neutralizing) monoclonal antibody, also reconstituted in 50% glycerol (employed for analyte labeling after conjugation with AF555 fluorescent dye) were from Toxin Technology (Sarasota, FL, USA). Strain B1409 of *E. coli* O157:H7 became available to our research center via a route of multiple destinations that last passed through the Centers for Disease Control and Prevention (Atlanta, GA, USA). Modified Brain Heart Infusion broth was from Becton Dickinson (Sparks, MD, USA). Any chemicals not mentioned were at least of reagent grade.

### 2.2. Apparatus

Solutions of biorecognition elements (antibodies in this manuscript) were orthogonally array printed into 96-well microtiter plate wells using an Omnigrid Accent Pro from Bucher (Basel, Switzerland) outfitted with a single, Stealth printing pin (model SMP3; TeleChem International, Inc., Sunnyvale, CA, USA). (Laser-induced fluorescence images were obtained with an LS400 scanner from Tecan Group Ltd. (Männedorf, Switzerland). Shaking of mictrotiter plates were conducted on a Titer Plate Shaker (Lab-Line Instruments, Inc.; Melrose Park, IL, USA) at slow-moderate speed setting. Microtiter plates were centrifuged in an Eppendorf refrigerated centrifuge (model 5810R) using an A-4-62 rotor (Eppendorf AG, Hamburg, Germany). Ultraviolet-Visible spectrophotometric readings were taken with a Cary 50 UV-Vis spectrophotometer (Varian, Inc., Palo Alto, CA, USA). Enumeration of intact bacterial cells was achieved with the aid of a Petroff-Hausser counting chamber obtained from Thomas Scientific (Swedesboro, NJ, USA).

### 2.3. Growth and Enumeration of Bacteria

Immediately prior to use, a frozen culture of stationary phase *E. coli* O157:H7 was thawed and added to modified Brain Heart Infusion broth (10 mL). This was incubated at 37 °C for 18 h with shaking at 160 rpm. Serial dilutions of cultures were enumerated using a Petroff-Hausser counting chamber as described by Gehring, *et al*. [29].

### 2.4. Conjugation of Antibodies with Fluorescent Dye

An AF555 dye labeling kit was used to prepare fluorescent BSA and antibody conjugates following the manufacturer’s instructions, briefly: BSA or antibody was diluted to ~1 mg/mL in 0.1 M carbonate buffer (pH 8.3), dye was added to ~0.5 mL of protein solution and incubated for 1 h at room temperature (RT) with stirring, the mix was eluted (using 10 mM PBS, pH 7.2 containing azide) through a gel filtration column to separate labeled protein from unbound dye and fractions of the first of two resolved colored bands were collected and pooled. The absorbance of pooled fractions was measured at 280 and 555 nm using a UV-Vis spectrophotometer in order to determine dye incorporation stoichiometry and antibody conjugate concentration.

### 2.5. Antibody Preparation and Microarray Printing

Biotinylated and non-biotinylated anti-*E. coli* O157:H7 capture antibodies (that were obtained as lyophilized reagents) were rehydrated in 50% glycerol to a concentration of 1 mg/mL that was further diluted to 1:30 in PBS containing 5% glycerol working solutions for microarray printing. (Glycerol was employed to prevent evaporation of the printed spots as well as to maintain hydration of the capture antibodies [30]). Anti-Stx antibodies were similarly reconstituted to 0.25 mg/mL in nH_2_O as directed by the supplier and further diluted 1:4 with PBS (containing 5% glycerol) for array printing.

Approximately 25 µL of capture antibody solution was pipetted into separate wells of a MicroAmp source plate on the microarray printer (positioned atop a 4 °C cooled thermal block during printing). Immediately prior to printing, source plates were centrifuged at 200× *g* for 2 min to remove any air bubbles. Array contact printing was performed with the following parameters—preprints/blots = 20; contact time = 0; dip and print acceleration = 10 cm/s^2^, and print velocity = 2 cm/s using an SMP3 (spot diameter of ~100 μm) pin that delivered ~0.7 nL per contact stroke. In each well, 2 columns of 8 spots per antibody were printed with a horizontal and vertical separation of 150 μm. After printing, all wells were visually examined, often with the assistance of a stereo light microscope (~10–20 × magnification) to ensure that spots were uniformly printed. Following array printing, spotted destination plates sat for 1 h at RT before use.

### 2.6. Antibody Microarray Detection of Bacteria and Shiga Toxin 1 in Multiwell Plates

**Figure 1:**
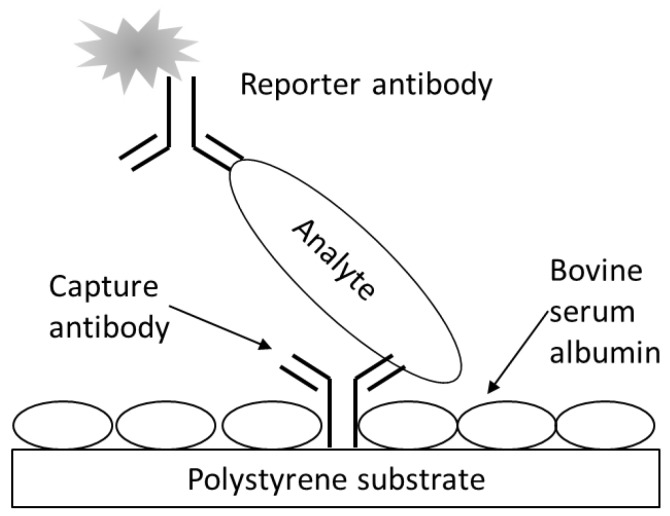
Fluorescence sandwich immunoassay schematic. Represented are analyte (e.g., bacterial cell or proteinaceous toxin) bound at a microarrayed spot of capture antibodies printing on the bottom of a well of a multiwell, black-walled, clear-bottomed, polystyrene microtiter plate. The bare polystyrene was further blocked with BSA and following analyte capture, reporter antibodies, conjugated with fluorescent molecules, were used to sandwich and label the analyte prior to detection with laser-induced fluorescence scanning.

A schematic for the fluorescence, sandwich immunoassay as applied to the multiwell antibody microarray-based detection *E. coli* O157 bacteria and Stx1 is depicted in Figure 1. The assay generally followed the one previously described for microarray slides [31] with several modifications. All immunoassay procedures and reagents were at RT. Wells of the destination plate, preprinted with capture antibody, were washed with 200 µL PBST (PBS containing 0.05% Tween 20), immediately emptied via rapid inversion of the plate, and any remaining liquid was removed by striking the plate (upside down) onto an absorbent towel laid flat on a laboratory bench. This wash procedure was repeated once with PBST. The plate wells were blocked with 50 µL of 1% BSA in PBS for 30 min. The plates were washed (as above) following removal of this BSA solution. Analyte (100 µL, or as indicated otherwise, of samples containing bacterial stock or Stx serially diluted in PBS) was then added, and each array was subjected to incubation (static, unless otherwise indicated) for 1 h (or time as otherwise indicated) to allow analyte capture. During the incubation for capture, the reporter antibody solutions were prepared (1:50 for AF555-labeled antibody conjugates) with PBST. The reporter antibodies were shielded from light during all experiments. The wells were washed twice with PBST and excess liquid was removed as above. Next, 50 μL reporter antibody solution was added to each well, which was subjected to static incubation for 1 h (unless otherwise indicated) at RT.

### 2.7. Scanning Electron Microscopy

After centrifugation concentration (3220× *g* for 5 min) of live *E. coli* O157:H7 cells in capture antibody (non-biotinylated) microarray-printed polystyrene microtiter plates, the cells fixed with 200 μL of 2.5% glutaraldehyde (Electron Microscopy Sciences, Hatfield, PA USA) for 30 min. The plates were then rinsed twice for 30 min each with ~200 μL per well of 0.1 M imidazole, (Electron Microscopy Sciences). The cell-associated bottoms (samples) of the wells were removed with a cork borer. The samples were then sequentially washed for 30 min intervals each with 2 mL of 50%, 80%, 90%, and finally 95% ethanol (The Warner-Graham Company, Cockeysville, MD, USA). The samples were momentarily held in and then washed with ~2 mL of 100% ethanol three times before being critically point dried. The samples were then stacked in a wire basket, separated by cloth, and placed in a Critical Point Drying Apparatus, (Denton Vacuum, Inc., Cherry Hill, NJ, USA), using liquid carbon dioxide (Welco Co, Allentown, PA, USA) for approximately 20 min. The samples were then removed and mounted on stubs and sputter gold coated for 30 s (EMS 150R ES, Electron Microscopy Sciences). They were then observed with Scanning Electron Microscope, FEI Quanta 200 F, (Hillsboro, OR, USA) with an accelerating voltage of 5–10 KV in high vacuum mode. It was observed that the critical point drying process shrunk the polystyrene discs to about 1/3 their original size.

### 2.8. Microarray Scanning and Data Analysis

Wells were washed twice with PBST and then were scanned at the appropriate fluorescence setting (AF555-excitation: 543 nm, emission filter: 590 nm) on the LS400 scanner using single channel scanning mode. During conjugate incubation, 543 and 643 nm lasers were turned on to warm up and stabilize for at least 30 min. Typical LS400 instrument scanning parameters, set and controlled via Array-Pro Analyzer software (ver. 4.5.1.73; Tecan Group Ltd., Männedorf, Switzerland) interface included: autofocusing in well mode, PMT gain that ranged from 100 to 150, 20 µm resolution, small pinhole setting, and optimization of integration time = 1. The multiwell plates had to be inverted during scanning.

Each well, which contained 8 printed spots per antibody, was considered an experimental unit. Fluorescence intensities (in fluorescence units or (arbitrary fluorescence units) AFUs) of sample spots and local/proximal (adjacent to sample spots in the same well) background pixels were obtained using the ArrayPro Software. Net spot intensities (sample responses minus individual, corresponding concentric and median background responses) were compared, and the 2 highest and 2 lowest values from each set of 8 were discarded. At least 3 technical replicates (single columns of 8 printed antibody spots in 3 individual microtiter plate wells) were generated for each concentration level of analyte tested. Some, but not all experiments were replicated over multiple days of experimentation. (No significant discrepancies between day-to-day replication were observed, data not shown.) Net intensities were then averaged and standard deviations, presented as error bars in plots, were computed for the means.

## 3. Results and Discussion

Maximizing binding of target is a critical factor in microarray detection. NeutrAvidin (NAv) is a deglycosylated form of avidin with a higher biotin binding specificity and lower non-specific binding. In a simple biotinylated globular protein (employing fluorescently labeled and biotinylated BSA that simulated biotinylated antibody) binding study, NAv, streptavidin (SAv), or biotinylated BSA (subsequently reacted with SAv) in different buffers were compared for capture efficacy of the dye-labeled protein following passive adsorption to polystyrene. Cursory results indicated that highest capture was with the NAv system, but the improvement was only marginal (~2×). Remarkably, direct adsorption of dye-labeled BSA elicited the same level of fluorescence as the SAv systems (data not shown). Direct adsorption of capture antibody to the well bottoms presented itself as an attractive and reasonably effective (with respect to fluorescence response) alternative, especially if the SAv/biotin binding system could be avoided altogether. Such passive adsorption of capture antibody serving as a foundation for fluorescence sandwich immunoassays was used throughout this study in conjunction with microarray detection (Figure 1).

**Figure 2:**
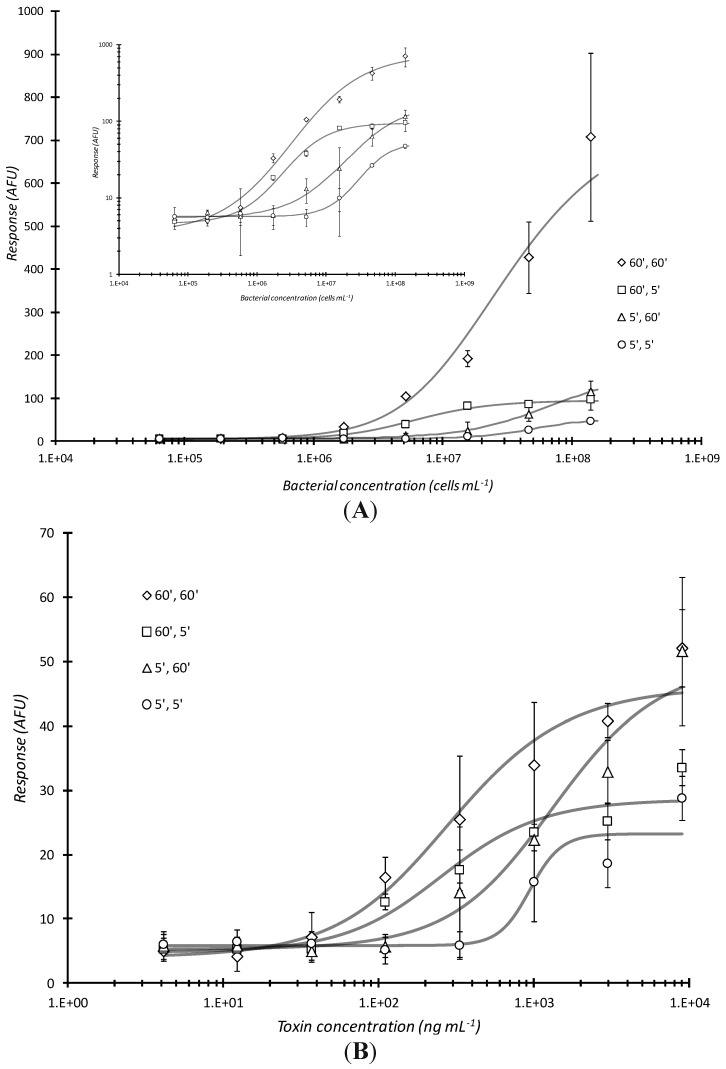
Effects of varying analyte and conjugation reaction times on microarray detection of bacterial cells or toxin. Serially diluted analyte, live *E. coli* O157:H7 (**A**) or Stx1 (**B**) were statically incubated in microtiter plates microarray printed with passively adsorbed biotinylated capture antibodies for the first time (5 or 60 min) indicated and then further reacted with fluorescent dye labeled antibody conjugates for the second time (5 or 60 min) also indicated. As with all subsequent plots in this report, the above curves show the microarray response in arbitrary fluorescence units (AFU) versus bacterial or toxin concentration. Each data point represented the mean ± standard deviation for 4 of 8 daily technical replicates (with 2 highest and 2 lowest values dropped) from serial dilution series combined with other similarly treated replicates from experiments repeated no less than 2 days and no more than 4 days.

The magnitude of microarray response was a function of the amount of time the analyte (bacteria or proteinaceous toxin) was in contact with the antibody arrayed plate well bottoms and that of the dwell time of the fluorescent antibody conjugate with captured analyte. The combination of 60 min analyte incubation with 60 min conjugate incubation, respectively, (or 60′, 60′) had the greatest response over all cell dilutions (Figure 2A). The next lowest plot is the 60′, 5′ (60 min sample incubation/capture, 5 min labeling antibody conjugate incubation; statistical difference P = 0.147, between these two curves was not significant) where the similar number of targets do not have the time to be detected by fluorescent antibody and the response curve saturates (particularly evident in the inset log-log plot in Figure 2A). For these and all subsequent results reported herein, a Student’s *t*-based statistical analysis was employed to test the homogeneity between regression coefficients of selected data sets [32]. Mass transport of reporter conjugate to the well surface tethered analyte appears to be a diffusion controlled, rate-limiting step in the assay. An even more interesting observation is between the 5′, 60′ and 60′, 5′ response curves (P = 0.00229). With the 5′, 60′ incubation, there were presumably fewer captured targets (bacterial analyte) for the fluorescent antibody to interact with as compared to 60′, 5′ where a concentration dependent curve became more evident. Since the 60′, 5′ curve exhibited greater sensitivity; this result indicated that the greater “bottleneck” was the analyte incubation time. The 5′, 5′ incubation conditions yielded the lowest response (P = 0.0408 *versus* 60′, 60′ and P = 0.000691 *vs*. 60′, 5′). Time of bacterial analyte contact was the determining factor for such static incubations. Using the same reaction conditions, similar, but much less profound trends were observed when the analyte was a proteinaceous toxin (Figure 2B). There was only a slight difference between the 60′, 60′ *vs*. 5′, 5′ curve (P = 0.0693) whereas there were no statistically significance differences between 60′, 60′ *vs*. 60′, 5′ (P = 0.433), 60′, 60′ *vs*. 5′, 60′ (P = 0.261), 60′, 5′ *vs*. 5′, 60′ (P = 0.623), 60′, 5′ *vs*. 5′, 5′ (P = 0.107), and 5′, 60′ *vs*. 5′, 5′ (P = 0.0753).

**Figure 3:**
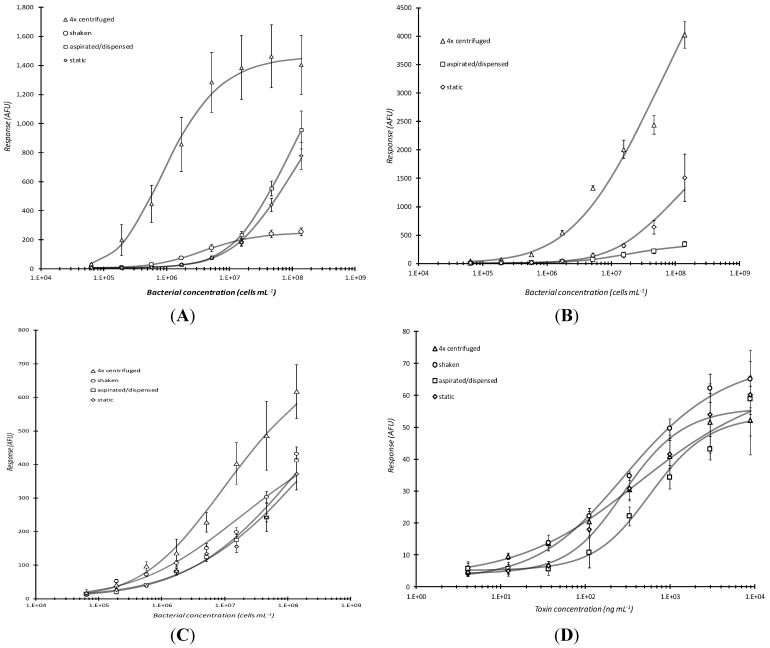

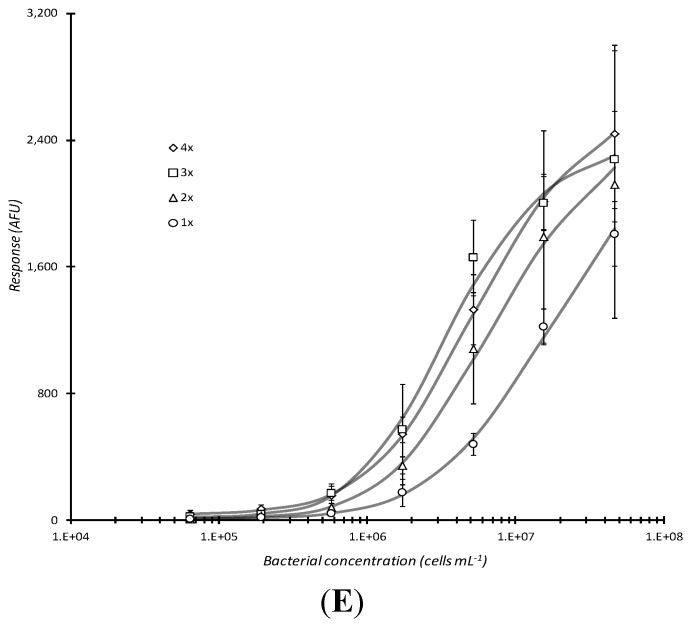
Comparison of incubation conditions in microarray detection of bacteria. Serially diluted analyte, live *E. coli* O157:H7, captured by passively adsorbed biotinylated (**A**) or non-biotinylated anti-*E. coli* O157:H7 antibody (**B**), heat-killed *E. coli* O157:H7 captured by biotinylated anti-O157:H7 antibody (**C**), or Stx1 captured with biotinylated anti-Stx1 antibody (**D**) were subjected to different incubation conditions during analyte incubation. Response *vs.* concentration curves are displayed above for static, aspirating/dispensing, shaking, and centrifugation incubation for 60 min at RT. The effect of multiple (1–4×), 5 min centrifugations (with resuspension of analyte mixture following each centrifugation) followed by static incubation for the remainder of 1 h total incubation time was also assessed for live *E. coli* O157:H7 that were reacted with non-biotinylated capture antibody (**E**). Each data point represented the mean ± standard deviation for 4 of 8 daily technical replicates (with 2 highest and 2 lowest values dropped) from serial dilution series combined with other similarly treated replicates from experiments repeated no less than 2 days and no more than 4 days.

Since bacterial analyte incubation time appeared to be the primary rate-limiting step during the immunoassay portion of plate-based microarray detection, various incubation treatments during analyte capture were tested to determine if detection could be improved relative to static incubation conditions (Figure 3). Three additional conditions were compared and they included: (1) shaking-moderate mixing speed on a platform shaker; (2) aspirating/dispensing-analyte mixtures were repeatedly (~3×) mixed manually via aspirating and dispensing with a multi-channel pipettor once every 5 min during the total analyte capture reaction time; and (3) centrifugation-analyte mixtures were added to the microtiter plate that was subsequently placed into a centrifuge outfitted with a swinging bucket rotor, the plate was spun for 5 min, and the mixture was aspirated/dispensed with a pipettor prior to additional incubation or a washing step.

It was not too surprising that centrifugation by far elicited the highest microarray response and best limit of detection for the live cells (Figure 3A,B) since bacterial cells are slightly denser than water. All of the curves for Figure 3A were statistically different except for “static” *vs*. “aspirated/dispensed.” (P = 0.0396 for 4× centrifuged *vs*. shaken; P = 0.0329 for 4× centrifuged *vs*. aspirated/dispensed; P = 0.0333 for 4× centrifuged *vs*. static; P = 0.00533 for shaken *vs*. aspirated/dispensed; P = 0.00704 for shaken *vs*. static; P = 0.317 for aspirated/dispensed *vs*. static.) Response levels were marginally (~2×) higher when non-biotinylated antibodies were used (Figure 3B). For Figure 3B, all of the curves were significantly different except again for “aspirated/dispensed” *vs*. “static.” (P = 0.000862, 4× centrifuged *vs*. aspirated/dispensed; P = 0.000942, 4× centrifuged *vs*. static; P = 0.174, aspirated/dispensed *vs*. static.) This result was reproducible and suggested that, as otherwise might be anticipated, a sub-population of the employed polyclonal antibodies had their antigen-binding sites deactivated (via steric hindrance and/or disruption of potential electrostatic interaction by amino acid functional groups) as a result of biotinylation. In other words, random, undirected biotinylation of antibodies may lower antibody specificity if epitope-binding site amines are blocked with biotin moieties.

Centrifugation had significantly less effect on heat-killed cells (Figure 3C; none of the curves in Figure 3C were significantly different with P ranging from 0.499 to 0.850) and, as would be expected with a relatively small biomolecular analyte, no considerable influence on the capture of the proteinaceous toxin Stx1 (Figure 3D) with P ranging from 0.130 to 0.587 expect for an unexpected difference between “aspirated/dispensed” *vs*. “static” (P = 0.0460). Upon visualization with light microscopy (data not shown), heat-killed *E. coli* O157:H7 cells have the appearance of disrupted cells fragmented into multiple pieces of various shapes, sizes, and density that has considerably more surface area available for binding by the polyclonal antibodies employed. Such fragments apparently do not share the same fluidic transport behavior observed with live (intact) cells.

Response after shaking was unexpectedly low for bacterial cells and even more surprisingly low with the multiple aspirating/dispensing technique (Figure 3A,B). It was hypothesized that continuous aspirating/dispensing would be analogous to improved capture typically observed in flow systems. However, it is possible that such action, and to a lesser extent, shaking, caused sheering of the bacteria from the surfaced of the antibody-coated well bottom/substrate. In addition, shaking possibly caused cells to be forced to the sides of the wells and therefore they did not interact with the printed antibodies on the bottom of the wells.

Repeated centrifugation of live cells did not appear to significantly improve binding to (non-biotinylated) capture antibody as observed by the marginal increase in microarray response (Figure 3E). Yet, upon statistical analysis, significant improvements were observed with 4× *vs*. 2× (P = 0.0477), 4× *vs*. 1× (P = 0.00339), 3× *vs*. 2× (P = 0.00673), 3× *vs*. 1× (P = 0.000453), and 2× *vs*. 1× (P = 0.0197), but not with 4× *vs*. 3× (P = 0.399). Together with the results observed for Stx1 dose-response (Figure 3D), this was evidence that the 60 min analyte incubation time could be considerably reduced upon substitution with a 5 min centrifugation step. Only slight improvement in microarray response for intact cells would be expected with additional centrifugation steps. Unfortunately, additional centrifugation would detrimentally add to the total assay time.

The assay conditions used for the generation of the 1x curve in Figure 3E were considered optimized and the final assay conditions for this investigation. A limit of detection of ~5.8 × 10^5^ cells/mL for live cells could be inferred from the 1x data set being a prospective lower detectable limit response value, minus its standard deviation, that was distinguishable from the baseline value at the lowest concentration plus 3× the standard deviation for that value.

**Figure 4:**
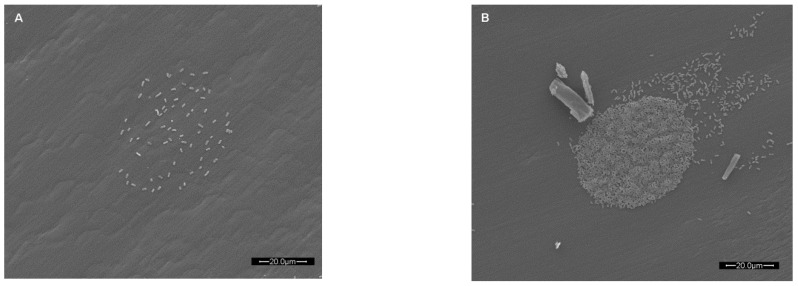
Scanning electron micrograph of bacteria associated with a single microarrayed spot of capture antibody. Live *E. coli* O157:H7, at two initial concentrations of 10^5^ CFU/mL (**A**) and 10^8^ CFU/mL (**B**) captured by array-printed antibodies (non-biotinylated anti-*E. coli* O157:H7 antibody) passively adsorbed to polystyrene well bottoms of a microtiter plate. Prior to SEM analysis, the BSA blocked plates were centrifuged (3220 × g for 5 min) to promote capture of bacteria (The scale bar is 20.0 µm in length).

Figure 4 displays scanning electron micrographs of fixed and dried *E. coli* O157:H7 cells captured by antibodies passively adsorbed to the well bottoms of polystyrene microtiter plates. Centrifugation was employed to enhance capture of the cells. Such an investigation may be used to correlate the fluorescence response versus the actual total number of cells associated with the microarrayed capture antibody spot. However, a very interesting observation was made that was particularly evident in Figure 4B. Whether array printing nucleic acids or antibodies, any excess unbound biorecognition element may bind outside of the intended printing area. Further binding of target (and subsequent label to the target) in these regions results in a smear often referred to as a “comet tailing”. Such comet tailing has been observed throughout years of our array-based research using various antibody systems at different concentration levels and washing techniques (data not shown). In Figure 4B, the comet tailing appears to represent excess capture antibody not thoroughly removed via washing and since washes were very rapid (<1 min) it thus provides evidence that adsorption of functionally active antibody onto “virgin” polystyrene was almost instantaneous.

**Figure 5:**
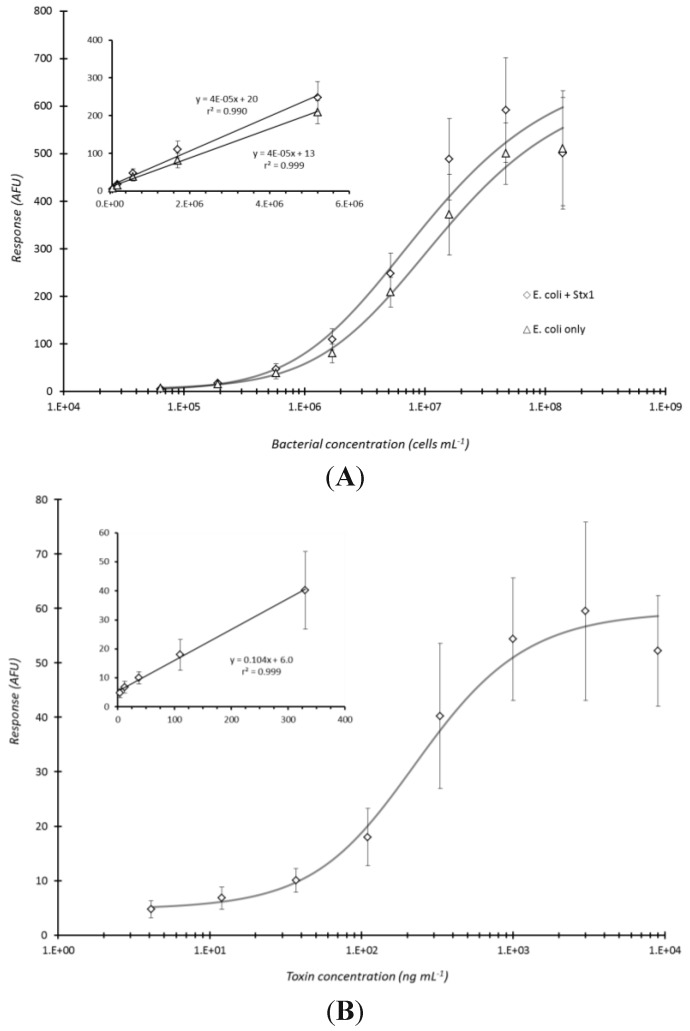
Microarray detection of bacteria and/or toxin under optimized immunoassay conditions. Heat-killed *E. coli* O157:H7 and Stx1 toxin were combined and 3-fold serially diluted in PBS to yield concentration ranges of 1.4 × 10^8^ to 6.4 × 10^4^ cells·mL^−1^ and 9 × 10^3^ to 4.1 ng·mL^−1^ for the bacteria and toxin, respectively. The samples were subjected to microarray detection using immunoassay conditions (sample centrifuged for 5 min with capture antibody (non-biotinylated for *E. coli* and biotinylated for toxin, microarray printed to the bottoms of microtiter plate wells) and then reacted with fluorescent antibody conjugate for 60 min before laser-induced fluorescence scanning) optimized in this investigation. The dose-response curves in (**A**) exhibit the microarray response versus concentration for *E. coli* O157:H7 in the presence or absence of Stx1 whereas (**B**) displays the dose-response curve for Stx1 in the presence of serially diluted *E. coli* O157:H7. Each data point represented the mean ± standard deviation for 4 of 8 daily technical replicates (with 2 highest and 2 lowest values dropped) from serial dilution series combined with other similarly treated replicates from experiments repeated once.

The established conditions (5 min centrifugation of analyte with array-printed antibodies and 60 min fluorescent antibody conjugate reaction) were considered an optimal compromise between immunoassay response and total assay time. The combination of these conditions was applied to the co-detection of *E. coli* O157:H7 bacterial cells and Stx1 toxin (Figure 5). Heat-killed cells were specifically selected for testing not only because the effect of centrifugation on bacterial analyte had already been assessed, but more so since it was of concern to task the assay (*i.e*., test for potential antibody cross-reactivity) with disrupted cells to insure that internal cell components would not affect the assay response when both analytes were combined for detection. Note, the selected Shigatoxigenic strain, B1409, of *E. coli* O157:H7 employed in this study produces only Stx2, and not Stx1. Figure 5A clearly shows that detection of the bacteria was essentially no different (P = 0.104) in the presence or absence of the toxin. The limit of detection, based on the criteria employed with Figure 3E (above), for the heat-killed cells (Figure 5A) was the same for the live cells being ~5.8 × 10^5^ cells/mL. Conversely, detection of Stx1, in the presence of the bacteria, exhibited essentially the same dose-response curve as observed for detection of toxin alone under the same conditions (refer to Figure 3D). The detection limit, also determined as above, for Stx1 was ~110 ng/mL as derived from the data presented in Figure 5B.

## 4. Conclusions

This investigation demonstrated that elimination of the costly streptavidin/biotin binding system with passive adsorption of microarray printed antibodies in 96-well, relatively inexpensive polystyrene microtiter plates can be a useful and cost-reduced method for high-throughput, multiplexed detection of analytes. This work further revealed that a 60 min static incubation may be replaced with a much shorter (5 min) centrifugation step that significantly increased detection response for intact bacteria (*E. coli* O157:H7) but not for a considerably smaller proteinaceous toxin (Stx1, a biomolecule of approx. molecular weight of 68 kD). Agitation of aqueous mixtures of analyte by shaking or manual aspiration/dispensing only marginally enhanced detection of live bacteria but had no impact on the detection of Stx1 indicating that intact cells, and not fragments or relatively small biomolecules, were solely influenced by the applied centrifugal force. A prospective alternative to centrifugation may be employing multiwell plates that incorporate biorecognition element arrayed filter membranes, a combination recently exhibited by [33]. Any process that forces analyte and antibody probe and/or subsequent reporter probe into close association will be advantageous to detection as exhibited by the method herein.

With the introduction of centrifugation during exposure of sample to capture antibody, a significant reduction in total assay time was afforded thus representing a major milestone towards the future development of an array-based assay that may be employed for typing mixed cultures within an 8 h workshift. This timeframe will allow for sample preparation (e.g., pre-filtration of extraneous matrix) and a brief growth enrichment culture that may be conducted in an MPN fashion if quantitation is desired. Therefore, intent was to limit the total immunoassay time to ~2.5 h, hence conditions that enhanced assay performance were primarily judged from signal amplitude, and secondarily for absolute error associated with individual data points. Though, as is often observed with immunoassay response curves, absolute error levels increased with analyte concentration, however, relative error generally remained constant. As described above, LOD determination only involved using background response and error as compared to near LOD response and error. Overall, this optimized assay yields conservatively determined limits of detection of 5.8 × 10^5^ cells/mL for both live and heat-killed *E. coli* O157:H7 and 110 ng/mL for Stx1in a total assay time of ~75 min. These results represent an ~40% improvement in bacterial detection limit for *E. coli* O157:H7 with a corresponding 50% reduction in total assay time as compared with an analogous assay previously developed by this group [15]. Though the results were promising, there is always room for improvement for this as well as other rapid methods since detection of “zero tolerance” pathogens ultimately requiring the need to detect a single cell in approx. 100 g or more of food.

In the future, these assays may incorporate automated plate handling, washing, and pipetting systems, as well as automated sample preparation for enhancement of sensitivity via target concentration achieved with cross-flow microfiltration [34] or antibody-coated paramagnetic particle-based immunomagnetic separation. This multiplex protein microarray format, performed in individual wells of 96-multiwell plates, may be used for high-throughput screening in clinical diagnostics and food testing as well as the characterization of biorecognition elements.
